# Students’ Scientific Society—How to Inspire Students and Help them to Become Oncologists? A Tutor’s Perspective

**DOI:** 10.1007/s13187-013-0587-5

**Published:** 2013-12-12

**Authors:** Radoslaw Tarkowski, Jan Kornafel

**Affiliations:** 1 Department of Oncology, Division of Surgical Oncology, Wroclaw Medical University, pl. Hirszfelda 12, 53-413 Wrocław, Poland; 2 Department of Oncology and Gynaecological Oncology Clinic, Wroclaw Medical University, pl. Hirszfelda 12, 53-413 Wrocław, Poland

## Background

The incidence of cancer continues to rise, and despite the development of prevention and treatment strategies, tumour burden still remains a major problem. Many may ask “who becomes an oncologist?” The answer is generally one who believes they can help and who believes in possibilities of oncologic treatment. Unfortunately, there are a large proportion of medical students who consider oncology as palliative care or at least a depressive branch of medicine which gives no hope and causes burn out syndrome rather quickly. Should we endeavour to change their point of view while studying at the medical universities? Of course, yes.

Many countries either risk or have a shortage of oncologists and this may be due, in part, to the negative view of oncology that is formed early on in medical school or past experience of losing a loved one to cancer. On the other hand, there are also students interested in the field of oncology but not yet convinced if they should pursue a career in this specialisation.

In 1988, the Department of Oncology of the Wroclaw Medical University (Poland) established a Students’ Scientific Society to provide an opportunity for medical students to learn more about oncology. There is evidence of similar activity published, showing its positive effect. Hendry et al. showed that such groups are not only supportive and socially cohesive but also increase the quality of their education. Regular meetings help to renew motivation for study, identify gaps in education and give explanations. Students taking part in such activities are self-regulating learners [1]. There are also problem-based learning (PBL) groups described in another publication [2]. Wood describes numerous advances of teamwork in such groups, i.e. the possibility of expanding communication skills and taking responsibility for learning. These are usually small group (counting 8–10) of students and a tutor, who should meet each other regularly and long enough to allow good and dynamic cooperation [2].

As a surgical oncologist working at the aforementioned department, I have tutored this group for the past 12 years. In this period, I have met many very interesting, intelligent, dedicated and creative students. Some of these perused careers in oncology, whilst others eventually chose other specialisations. However, all of them learned about oncology by taking part in our activity more than they were obliged during regular lectures and seminars. Whilst not all of them became oncologists, all will encounter cancer patients throughout their careers.

## Objective

The objective of this manuscript is to show a role of activity in Students’ Scientific Society in enhancing motivation for expanding the interest in oncology, promoting self-learning, teamwork and building skills valuable in future professional career as a doctor.

## Methods

There are usually around 40 students registered in the group each academic year. The participants are recruited from the Faculty of Medicine, which counts 1,690 students (global number of all students of all 6 years). There were single participants from the other faculties: one from the Faculty of Health Science and two from the Department of Medical Laboratory Diagnostic, a part of the Faculty of Pharmacy. All three of them were very active despite of some differences in the programme of the studies. As mentioned above, medical studies at the Faculty of Medicine last 6 years in our country. There are basic sciences which are the core of the teaching programme during first 3 years, although propaedeutics of oncology and propaedeutics of internal medicine appear in the beginning of the third year. Perhaps, this is the reason why there are many third year students becoming participants in this moment of their education. Last 3 years are focused on clinical medicine. Internship lasting 13 months follows the studies and does not include obligatory training in oncology within. Available records concerning the number of the participants start from 1999 and show total number of 450 persons. Freshmen are absent due to engagement in anatomy and other basic sciences. Students join from the second year of studies, at which time they usually improve their skills in understanding basic sciences as well as being very eager to encounter some clinical and practical activity. There are also students in the sixth year, who have just touched the core of the oncologic programme. Finally, I meet some alumni as well, the former active members of our society who become residents. This diversity means that the programme of our meetings should be well balanced and interesting to all participants in this rather heterogenic group.

We usually meet once per month, and the meeting last about 2 h. There are also consultations with the tutor, especially before major conferences and presentations.

Participation in the society is voluntary—they are not obliged to take part in activities of the society due to study regulations. Of course, there are some official and known benefits (i.e. points in Erasmus programme score and enabling studying abroad in frames of the students’ exchange programme). Most participate to improve their knowledge and skills. Students want to experience what oncology is like and what does it mean to be oncologist. They look for their way in the future by means of medical specialty. When I ask the students about their choice and special interests, many of them are undecided, even when they are going to finish their studies. Whilst in the society, they will be exposed to surgeons, medical oncologist and radiation oncologists and have the opportunity to build upon the knowledge gained throughout their education programme (i.e. taking part in operations, small group tutorials, participating in clinics and attending workshops and other ‘ad hoc’ learning opportunities). Sometimes, we discuss issues with a clinical psychologist, since the need of psychological support and intervention is very important, but often disregarded both by patients and physicians. Some students will find oncology to be a most interesting specialisation. This is clear to see when I meet former members of the scientific society, who are working as doctors at the same cancer centre.

This is a practical aspect of our activity. The other—very important—is the scientific one, as it stands in the name of the society. Students have some questions and ideas, and here, they can expand them by constructing and conducting studies. They receive help from my colleagues and me. Due to close cooperation with our regional cancer centre, we have permission for our students to have access to patients and their medical records. Since the person of the patient is a core of our activity, I prefer studies concerning psychological issues, adverse effects of therapy and their impact on quality of life. It concerns not only patients and potential patients but also health care professionals and medical students as well. The opportunity of meeting patients with cancer seems to be very attractive not only for last year students (who will soon face many practical problems and wish to be prepared for such encounters) but also more junior students studying mainly basic sciences. They have the opportunity to apply theory to the cases they see and offer practical solutions during our meetings. They appreciate joining science with practice.

Once the studies are completed, students present them during conferences for students and young scientists. Some of them have been presented during oncologist’s conferences in an ‘open’ category. Our students have won prizes, both for oral presentations and scientific posters presented on both the domestic and international stages. This kind of activity is very stimulating, and the prizes are motivating. They are important steps in their professional career. The word presentation is crucial in this part of the text. After becoming doctors, today, students will present—cases, studies, reviews, etc., sooner or later. The art of proper presentation enables clear and effective communication of one’s message. Here, they can learn how to present and how to speak in public. Such training in front of other students seems to be far less stressful than a lecture or presentation in front of the head of department or senior colleagues or in front of a large audience, full of professionals during a scientific conference. Some of us are born actors, some are very shy. There are also shy actors as well but well trained and thus rewarded. Here, students have an opportunity to train. I asked my friend, who is a soldier (ranger), if he was always ready to face dangerous tasks and activities or whether he was born to feel no fear. He told me that the rangers have been selected due to some obvious predispositions for such special acting, but there is also training that makes them who they are. So, if you are scared, you have to break the barrier (usually a mental one). So students ‘jump from the cliff’ and become interesting and effective presenters. Usually, I ask several people to prepare a topic, a review or presentation of the article and show it during our meeting. They stand up and present and I can see that it works! Such experience helps students a lot. Students do not limit their activity to presenting studies during scientific events. They also organize such events (usually addressed to other students), playing the main role in inviting speakers, constructing the scientific programme and finding financial resources for catering, posters, invitations, etc. They are really well organized.

There is another important part of our activity: the social one. Students meet each other, prepare presentations and learn how to work together in team. Usually, we organize a trip once a year to a nice and interesting place, such as a village in the mountains or one of the European cultural capitals (i.e. Krakow, Prague or Berlin). We discuss oncological topics, appreciate the local culture and enjoy each other’s company. We meet new friends and delight in the camaraderie of teamwork and importantly, we get some rest.

This is a very important part of my duty as a lecturer, and I enjoy it very much. I am very glad and very honoured to work with these students, who are filled with so much enthusiasm. Seeing new oncologists, who were once students of the Scientific Society, is the best confirmation of the effectiveness of such an activity and one that is extremely gratifying.
